# Cloning, Expression, and Purification of a Nitric Oxide Synthase-Like Protein from *Bacillus cereus*

**DOI:** 10.1155/2010/489892

**Published:** 2009-11-30

**Authors:** Heather J. Montgomery, Andrea L. Dupont, Hilary E. Leivo, J. Guy Guillemette

**Affiliations:** Department of Chemistry, University of Waterloo, Waterloo, ON, Canada N2L 3G1

## Abstract

The nitric oxide synthase-like protein from *Bacillus cereus* (bcNOS) has been cloned, expressed, and characterized. This small hemeprotein (356 amino acids in length) has a mass of 43 kDa and forms a dimer. The recombinant protein showed similar spectral shifts to the mammalian NOS proteins and could bind the substrates L-arginine and N^G^-hydroxy-L-arginine as well as the ligand imidazole. Low levels of activity were recorded for the hydrogen peroxide-dependent oxidation of N^G^-hydroxy-L-arginine and L-arginine by bcNOS, while a reconstituted system with the rat neuronal NOS reductase domain showed no activity. The recombinant bcNOS protein adds to the complement of bacterial NOS-like proteins that are used for the investigation of the mechanism and function of NO in microorganisms.

## 1. Introduction

Nitric oxide has many diverse functions in the mammalian body and is produced in mammals by a family of nitric oxide synthase enzymes (NOS: EC 1.14.13.39) [1]. These enzymes are composed of an N-terminus oxygenase domain containing a heme, tetrahydrobiopterin (H_4_B), and the substrate L-arginine as well as a C-terminus reductase domain. There are over thirty NOS-like proteins in prokaryotes including eight different types reported in *bacilli *[2]. Bacterial NOS-like enzymes lack the associated NOS reductase domain found in mammalian enzymes [3]. The bacterial NOSs have many properties in common with the mammalian oxygenase domain including dimer structure, L-Arginine as a substrate, and typical heme spectroscopy [2]. Very little is known about the function of these proteins in prokaryotes, and they are not required for nitrification and denitrification pathways [4]. 

The opportunistic pathogen *B. cereus *causes food poisoning and is closely related to the animal and human pathogen *B. anthracis *used as a biological weapon as well as the insect pathogen *B. thuringiensis *that is used as a pesticide. We report the cloning, expression, purification, and characterization of a NOS-like protein from *Bacillus cereus* (bcNOS).

## 2. Materials and Methods

### 2.1. Materials

All reagents were purchased from Sigma-Adrich Canada Ltd. (Oakville, ON, Canada) and Fisher-Scientific Ltd. (Ottawa, ON, Canada) and were of high quality chemical grade.

### 2.2. Molecular Biology

The bcNOS gene from *B*. *cereus *(ATCC strain number 10987) was amplified by PCR from genomic DNA. The following PCR primers generated an Nde I site (bold) before the 5′ start codon in BCNOSF1 and an EcoR I site (underlined) after the 3′ stop codon in BCNOSR1: BCNOSF1, 5′-GAA GAT CT**C ATA TG**A GTA AAA CGA AGC AAT TAA TAG AGG AAG CG-3′; BCNOSREV, 5′-GGG AAT TCC TAT TTA TGA AAA AAA TTC GGC TTC AAA ATT TC-3′. The amplified fragment was cloned into the pET28a expression vector (Novagen, Madison, WI), which contains a His_6 _coding region upstream from the Nde I site.

### 2.3. Expression and Purification of Proteins

*E. coli* strain BL21(DE3) pLysS transfected with the bcNOS plasmid and grown in Luria-Bertani broth containing 100 *μ*g/mL ampicillin, induced at an OD (600 nm) of 0.6 with 1 mM IPTG and grown for 4 hours and then harvested. The recombinant bcNOS was purified from the bacterial cells using metal chelating chromatography [5] (see Supplementary Material available online at doi: 10.1155/2010/489892). Recombinant rat nNOS reductase protein was overexpressed in *Escherichia coli *strain BL21(DE3) and purified as previously described [6].

### 2.4. Mass Spectrometry and Chromatography

Mass spectrometry of the purified bcNOS was performed at the WATSPEC Mass Spectroscopy Facility at the University of Waterloo [5]. Gel exclusion chromatography was used to estimate the molecular weight of bcNOS dimer [5].

### 2.5. Spectroscopy

Spectroscopy performed on a Varian-Cary 1. The bcNOS exhibited a typical heme peak around 400 nm. Enzyme (10 *μ*M) was incubated in the presence of 1 mM imidazole, 1 mM L-arginine, 100 *μ*M H_4_B, or CO gas. Difference spectroscopy was used to measure the binding affinities of imidazole, NOHA, and L-arginine as previously described [7].

### 2.6. Catalytic Activity Assays

The H_2_O_2_-dependent bcNOS oxidation of NOHA and L-arginine to nitrite was monitored at 25°C on a 96-well plate reader as presiously reported [8, 9]. The hemoglobin capture assay [10] was used to monitor nitric oxide production from bcNOS in the presence of L-arginine and the rat neuronal NOS reductase domain.

## 3. Results and Discussion

The sequence alignment of the bcNOS protein with the NOS-like proteins from *B. subtilis *(bsNOS), *Staphylococcus aureus *(saNOS), and *D. radiodurans *(deiNOS) revealed a 42 to 52% identity and 61 to 68% conservation of the sequence and full conservation of the residues involved in binding to the heme (see Figure 1 in supplementary materials). A comparison of the bcNOS protein with the human inducible NOS (iNOS) revealed a 40% identity and 57% conservation of the sequence. Recombinant bcNOS was purified to greater than 90% homogeneity based upon SDS-PAGE and mass spectrometry analysis (see Figure 2 in supplementary materials). A yield of 8 mg of pure bcNOS was obtained per liter of culture. Nondenaturing native-PAGE and gel exclusion chromatography both showed that bcNOS forms a dimer. 

**Figure 1:**
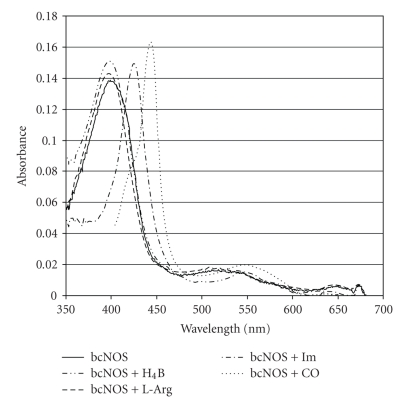
Spectral graphs of bcNOS with heme bind ligands. The UV-visible spectrum of bcNOS (—) protein was performed as stated in the Materials and Methods. Protein was incubated in the presence of H_4_B (- ^. .^ -), L-arginine (- - -), imidazole (- ^.^ -), and CO (^. . . .^).

The bcNOS UV-visible spectrum showed the presence of a heme chromophore in a high spin state (Figure 1). The observed spectral changes of bcNOS due to the binding of different heme ligands are summarized in Table 1. The characteristic Soret absorbance peak of 399 nm was observed for ferric bcNOS (Figure 1), likely corresponding to a mixture of 5-coordinated high-spin species and a water-bound 6-coordinated low-spin species. The binding of DDT shifts the equilibrium towards a fully 5-coordinated high-spin species. When H_4_B was added the Soret peak shifted to 397 nm. The slight blue shift in the spectrum indicates that the H_4_B displaced the DTT ligand. Adding L-arginine produced the same result as H_4_B, resulting in a Soret absorbance peak shift to 397 nm. The addition of imidazole resulted in a low-spin heme state with a peak absorbance of 426 nm that is typical of imidazole serving as a distal sixth ligand in the active site of NOS enzymes. Dithionite-reduced, carbon monoxide (CO) bound bcNOS gave absorbance peaks at 443 nm and 545 nm, comparable to the mammalian iNOS and neuronal NOS (nNOS) oxygenase domains and other bacterial NOS-like proteins (Table 1) indicating a similar heme iron coordination structure. 

Difference spectrometry, used to study heme-substrate interactions, revealed that a substrate-binding site exists in bcNOS close to the heme group and that this site is capable of binding imidazole, NOHA, or L-arginine. The displacement of heme-bound imidazole due to the binding of NOHA or L-arginine was monitored to determine the binding affinity of NOHA and L-arginine to bcNOS. Double-reciprocal analysis of the binding of imidazole to bcNOS gave a *K*_*d*_ value of 181 ± 13 *μ*M. The binding affinities of NOHA and L-arginine were much greater with *K*_*s*_ values of 1.12 ± 0.01 and 11 ± 3, respectively. The apparent binding affinity of L-arginine to bcNOS was found to be similar to those determined for other bacterial NOS proteins and the mammalian iNOSoxy protein (Table 2). This is consistent with bcNOS containing a conserved glutamate residue found to be essential for high affinity L-arginine binding in mammalian NOS enzymes [13]. 

Tetrahydrobiopterin-free nNOS can oxidize NOHA or L-arginine to nitrite in the presence of H_2_O_2_ [14]. Despite low binding constants for both NOHA and L-arginine, low levels of activity were found for the bcNOS protein in the presence of H_2_O_2_ and either NOHA (0.100 ± 0.014 min^−1^) or L-arginine (0.048 ± 0.001 minute). When compared to mammalian NOS enzymes, slow turnover numbers for H_2_O_2_-supported NOHA oxidation have also been reported for other bacterial NOS-like proteins [11, 15, 16]. The addition of H_4_B did not significantly increase the activity of bcNOS, but in the presence of NOHA and THF the activity doubled (0.231 ± 0.023). This suggests that a biological ligand, such as THF or a related pterin, may be required by bcNOS for catalytic activity. A similar catalytic rate was determined for saNOS in the absence of a cofactor (0.15 ± 0.01 nmol nitrite min^−1^nmol saNOS) with no increase in activity observed in the presence of THF [17] and a recent report indicates that THF may replace H_4_B as a redox-active cofactor in deiNOS [15]. 

The bcNOS protein was not catalytically active when coupled with the rat nNOS reductase domain protein in the presence of L-arginine and NADPH despite the fact that the nNOS reductase domain protein readily transfers electrons to reduce cytochrome *c *[6]. The relevance of such a reconstituted system comes into question, as a reductase protein similar to the mammalian NOS reductase domain could not be found in the *B. cereus *genome. Bacterial flavodoxins have been reported to support nitric oxide production by *B. subtilis* nitric oxide synthase [16]. We were unable to identify the electron donor(s) of bcNOS which is consistent with a recent report showing that bacterial NOS proteins do not appear to accept electrons from a specific reductase but more likely accept electrons from several different sources [18]. 

The NO produced by bacterial NOS enzymes has been associated with a number of novel functions. In *Streptomyces *NOS mediates the nitration of the tryptophan moiety of the phytotoxin dipeptide L-tryptophan-L-phenylalanine [19]. Disruption of the NOS gene in *B. subtilis* renders the strain more susceptible to oxidative damage [20]. Our *B. cereus nos*gene knock out strain was also found to be more vulnerable to hydrogen peroxide exposure (results not shown). Notably, *B. anthracis *derived-NO is correlated with pathogen virulence and survival in macrophages [21]. Clearly, there are several unanswered questions regarding bacterial NOS enzymes including cofactor requirements, evolutionary traits, catalytic mechanism, their biological reductase partners, and their in vivo function(s).

## Supplementary Material

The supplementary material includes sequence alignments of bacterial NOS-like proteins and three mammalian NOS oxygenase domains. The results of our complete characterization of the the *Bacillus cereus* NOS-like protein are also included in the supplementary section.

## Figures and Tables

**Table 1:** Soret and visible spectral properties (wavelength (nm) at peak absorbance) of bcNOS, deiNOS, bsNOS, nNOSoxy, and iNOSoxy with L-arginine and other various heme ligands.

Enzyme-ligand complex	bcNOS		deiNOS^a^		bsNOS^b^	nNOSoxy^a^		iNOSoxy^b^
	Soret	Visible	Soret	Visible	Soret	Soret	Visible	Soret
Ferric enzyme	399	650	N.D.	N.D.	402	N.D.	N.D.	418
+ H_4_B	397	650	N.D.	N.D.	399	N.D.	N.D.	400
+ L-Arg	397	645	393	650	398	393	650	401
+ L-Arg + H_4_B	395	643	N.D.	N.D.	395	N.D.	N.D.	396
+ Imidazole	426	547	427	550	426	427	550	427
+ Dithiothreitol	399	650	380, 460	650	400	380, 460	650	375, 459
Ferrous-CO	443	545	444	540	445	444	540	444

^a^taken from [11]. ^b^taken from [12]. N.D.: not determined. nNOSoxy: neuronal NOS oxygenase domain. iNOSoxy: inducible NOS oxygenase domain.

**Table 2:** Comparison of the binding properties of bcNOS to other bacterial NOS-like and mammalian NOS oxygenase domain proteins.

Binding constants (*μ*M)	bcNOS	bsNOS^a^	deiNOS^b^	iNOSoxy^a^	nNOSoxy^b^
*K*_*d*_ imidazole	181 ± 13	384 ± 10	—	158 ± 6	—
*K*_obs_ L-Arg	596 ± 161	129 ± 2	97 ± 10	175 ± 4	55 ± 4
*K*_s_ L-Arg	11 ± 3	4.8 ± 0.1	—	16.1 ± 0.7	—
*K*_s_ NOHA	1.12 ± 0.01	—	—	—	—

^a^taken from [8]. ^b^taken from [11]. iNOSoxy: inducible NOS oxygenase domain. nNOSoxy: neuronal NOS oxygenase domain.

## Abbreviations

*ba:*: *Bacillus anthracis*
*bs:*: *Bacillus subtilis*
dei: *D. radiodurans*
H_4_B: (6R)-5,6,7,8-tetrahydrobiopterin
NOHA: N^G^-hydroxy-L-arginine
NOS: Nitric oxide synthase
NOS_oxy_: Oxygenase domain of NOS
Sa: *Staphylococcus aureus*
THF: Yetrahydrofolate.

## References

[1] Nathan C (1992). Nitric oxide as a secretory product of mammalian cells. *The FASEB Journal*.

[2] Sudhamsu J, Crane BR (2009). Bacterial nitric oxide synthases: what are they good for?. *Trends in Microbiology*.

[3] Crane BR (2008). The enzymology of nitric oxide in bacterial pathogenesis and resistance. *Biochemical Society Transactions*.

[4] Goretski J, Hollocher TC (1990). The kinetic and isotopic competence of nitric oxide as an intermediate in denitrification. *The Journal of Biological Chemistry*.

[5] Spratt DE, Newman E, Mosher J, Ghosh DK, Salerno JC, Guillemette JG (2006). Binding and activation of nitric oxide synthase isozymes by calmodulin EF hand pairs. *FEBS Journal*.

[6] Montgomery HJ, Romanov V, Guillemette JG (2000). Removal of a putative inhibitory element reduces the calcium-dependent calmodulin activation of neuronal nitric-oxide synthase. *The Journal of Biological Chemistry*.

[7] McMillan K, Masters BSS (1993). Optical difference spectrophotometry as a probe of rat brain nitric oxide synthase heme-substrate interaction. *Biochemistry*.

[8] Choi W-S, Chang M-S, Han J-W, Hong S-Y, Lee H-W (1997). Identification of nitric oxide synthase in *Staphylococcus aureus*. *Biochemical and Biophysical Research Communications*.

[9] Perdicakis B, Montgomery HJ, Abbott GL (2005). Photocontrol of nitric oxide production in cell culture using a caged isoform selective inhibitor. *Bioorganic and Medicinal Chemistry*.

[10] Montgomery HJ, Bartlett R, Perdicakis B, Jervis E, Squier TC, Guillemette JG (2003). Activation of constitutive nitric oxide synthases by oxidized calmodulin mutants. *Biochemistry*.

[11] Adak S, Bilwes AM, Panda K (2002). Cloning, expression, and characterization of a nitric oxide synthase protein from *Deinococcus radiodurans*. *Proceedings of the National Academy of Sciences of the United States of America*.

[12] Wang Z-Q, Wei C-C, Sharma M, Pant K, Crane BR, Stuehr DJ (2004). A conserved Val to Ile switch near the heme pocket of animal and bacterial nitric-oxide synthases helps determine their distinct catalytic profiles. *The Journal of Biological Chemistry*.

[13] Gachhui R, Ghosh DK, Wu C, Parkinson J, Crane BR, Stuehr DJ (1997). Mutagenesis of acidic residues in the oxygenase domain of inducible nitric-oxide synthase identifies a glutamate involved in arginine binding. *Biochemistry*.

[14] Adak S, Wang Q, Stuehr DJ (2000). Arginine conversion to nitroxide by tetrahydrobiopterin-free neuronal nitric-oxide synthase: implications for mechanism. *The Journal of Biological Chemistry*.

[15] Reece SY, Woodward JJ, Marletta MA (2009). Synthesis of nitric oxide by the NOS-like protein from *Deinococcus radiodurans*: a direct role for tetrahydrofolate. *Biochemistry*.

[16] Wang Z-Q, Lawson RJ, Buddha MR (2007). Bacterial flavodoxins support nitric oxide production by *Bacillus subtilis* nitric-oxide synthase. *The Journal of Biological Chemistry*.

[17] Presta A, Siddhanta U, Wu C (1998). Comparative functioning of dihydro- and tetrahydropterins in supporting electron transfer, catalysis, and subunit dimerization in inducible nitric oxide synthase. *Biochemistry*.

[18] Gusarov I, Starodubtseva M, Wang Z-Q (2008). Bacterial nitric-oxide synthases operate without a dedicated redox partner. *The Journal of Biological Chemistry*.

[19] Kers JA, Wach MJ, Krasnoff SU (2004). Nitration of a peptide phytotoxin by bacterial nitric oxide synthase. *Nature*.

[20] Gusarov I, Nudler E (2005). NO-mediated cytoprotection: instant adaptation to oxidative stress in bacteria. *Proceedings of the National Academy of Sciences of the United States of America*.

[21] Shatalin K, Gusarov I, Avetissova E (2008). Bacillus anthracis-derived nitric oxide is essential for pathogen virulence and survival in macrophages. *Proceedings of the National Academy of Sciences of the United States of America*.

