# Supplementary material for: Cloning, Expression, and Purification of a Nitric Oxide Synthase-Like Protein from Bacillus cereus
Source: Biochem Res Int. 2009 Nov 30;2010:489892. doi: 10.1155/2010/489892 (PMC3005942; doi:10.1155/2010/489892)
Supplement: Supplementary file 1 — The supplementary material includes sequence alignments of bacterial NOS-like proteins and three mammalian NOS oxygenase domains. The results of our complete characterization of the the Bacillus cereus NOS-like protein are also included in the supplementary section. [file 489892.f1.pdf]

```
bcNOS    1 : --------------------------------------------------MSKTKQLIEEASHFITICYKEL-NK
bsNOS    - : ---------------------------------------------------------------------------
SANOS    1 : -------------------------------------------------------MLFKEAQAFIENMYKEC-HY
deiNOS   1 : -------------------------------------------------------MSCPAAAVLTPDMRAFLRRF
miNOS   77 : QYVRIKNWGSGEILHDTLHHKATSDFTCKSKSCLGSIMNPKSLTRGPRDKPTPLEELLPHAIEFINQYYGSFKEA
hnNOS  304 : RFLKVKNWETEVVLTDTLHLKSTLETGCTEYICMGSIMHPSQHARRPE-DVRTKGQLFPLAKEFIDQYYSSIKRF
beNOS   69 : KFPRVKNWELGSITYDTLCAQSQQDGPCTPRCCLGSLVLPRKLQTRPSPGPPPAEQLLSQARDFINQYYSSIKRS

bcNOS   25 : E-QFIEERMKEIRVEIEKTGTYEHTFEELVHGSRMAWRNSNRCIGRLFWSKMHILDAREVNDEEGVYNALIHHIK
bsNOS    1 : ----MKDRLADIKSEIDLTGSYVHTKEELEHGAKMAWRNSNRCIGRLFWNSLNVIDRRDVRTKEEVRDALFHHIE
SANOS   20 : ETQIINKRLHDIELEIKETGTYTHTEEELIYGAKMAWRNSNRCIGRLFWDSLNVIDARDVTDEASFLSSITYHIT
deiNOS  21 : HEEMGEPGLPARLRAVEEAGLWWPTSAELTWGAKVAWRNSTRCVGRLYWEALSVRDLRELNTAQAVYEALLQHLD
miNOS  152 : KIEEHLARLEAVTKEIETTGTYQLTLDELIFATKMAWRNAPRCIGRIQWSNLQVFDARNCSTAQEMFQHICRHIL
hnNOS  378 : GSKAHMERLEEVNKEIDTTSTYQLKDTELIYGAKHAWRNASRCVGRIQWSKLQVFDARDCTTAHGMFNYICNHVK
beNOS  144 : GSQAHEERLQEVEAEVASTGTYHLRESELVFGAKQAWRNAPRCVGRIQWGKLQVFDARDCSSAQEMFTYICNHIK

bcNOS   99 : YATNDGKVKPTITIFKQYQGEENNIRIYNHQLIRYAGYKTE-MGVIGDSHSAVFTDFCQELGWQGEGTNFDVLPL
bsNOS   72 : TATNNGKIRPTITIFPPEEKGEKQVEIWNHQLIRYAGYESD-GERIGDPASCSLTAACEELGWRGERTDFDLLPL
SANOS   95 : QATNEGKLKPYITIYAPKDGP----KIFNNQLIRYAGYDN-----CGDPAEKEVTRLANHLGWKGKGTNFDVLPL
deiNOS  96 : DAFCGGHIRPVISVFGP------GVRLHNPQLIRYA----------DDPINADFVDKLRRFGWQPRGERFEVLPL
miNOS  227 : YATNNGNIRSAITVFPQRSDGKHDFRLWNSQLIRYAGYQMPDGTIRGDAATLEFTQLCIDLGWKPRYGRFDVLPL
hnNOS  453 : YATNKGNLRSAITIFPQRTDGKHDFRVWNSQLIRYAGYKQPDGSTLGDPANVQFTEICIQQGWKPPRGRFDVLPL
beNOS  219 : YATNRGNLRSAITVFPQRAPGRGDFRIWNSQLVRYAGYRQQDGSVRGDPANVEITELCIQHGWTPGNGRFDVLPL

bcNOS  173 : VFSIDG-KAPIYKEIPKEEVKEVPIEHPEYP-ISSLGAKWYGVPMISDMRLEIGGISYTAAPFNGWYMGTEIGAR
bsNOS  146 : IFRMKGDEQPVWYELPRSLVIEVPITHPDIEAFSDLELKWYGVPIISDMKLEVGGIHYNAAPFNGWYMGTEIGAR
SANOS  161 : IYQLPN-ESVKFYEYPTSLIKEVPIEHNHYPKLRKLNLKWYAVPIISNMDLKIGGIVYPTAPFNGWYMVTEIGVR
deiNOS 155 : LIEVNG--RAELFSLPPQAVQEVAITHPVCLGIGELGLRWHALPVISDMHLDIGGLHLP-CAFSGWYVQTEIAAR
miNOS  302 : VLQADG-QDPEVFEIPPDLVLEVTMEHPKYEWFQELGLKWYALPAVANMLLEVGGLEFPACPFNGWYMGTEIGVR
hnNOS  528 : LLQANG-NDPELFQIPPELVLEVPIRHPKFEWFKDLGLKWYGLPAVSNMLLEIGGLEFSACPFSGWYMGTEIGVR
beNOS  294 : LLQAPD-EAPELFVLPPELVLEVPLEHPTLEWFAALGLRWYALPAVSNMLLEIGGLEFSAAPFSGWYMSTEIGTR

bcNOS  246 : NLADHDRYNLLPAVAEMMDLDTSRNGTLWKDKALVELNVAVLHSFKKQGVSIVDHHTAAQQFQQFEKQEAACGRV
bsNOS  221 : NLADEKRYDKLKKVASVIGIAADYNTDLWKDQALVELNKAVLHSYKKQGVSIVDHHTAASQFKRFEEQEEEAGRK
SANOS  235 : NFIDDYRYNLLEKVADAFEFDTLKNNSFNKDRALVELNYAVYHSFKKEGVSIVDHLTAAKQFELFERNEAQQGRQ
deiNOS 227 : DLADVGRYDQLPAVARALGLDTSRERTLWRDRALVELNVAVLHSFDAAGVKLADHHTVTAHHVRFEERARAGRE
miNOS  376 : DFCDTQRYNILEEVGRRMGLETHTLASLWKDRAVTEINVAVLHSFQKQNVTIMDHHTASESFMKHMQNEYRARGG
hnNOS  602 : DYCDNSRYNILEEVAKKMNLDMRKTSSLWKDQALVEINIAVLYSFQSDKVTIVDHHSATESFIKHMENEYRCRGG
beNOS  368 : NLCDPHRYNILEDVAVCMDLDTRTTSSLWKDKAAVEINLAVLHSFQLAKVTIVDHHAATVSFMKHLDNEQKARGG

bcNOS  321 : VTGNWVWLIPPLSPATTHIYHKPYPNEILKPNFFHK---------------------------------------
bsNOS  296 : LTGDWTWLIPPISPAATHIFHRSYDNSIVKPNYFYQDKPYE----------------------------------
SANOS  310 : VTGKWSWLAPPLSPTLTSNYHHGYDNTVKDPNFFYKKKESNANQCPFHH--------------------------
deiNOS 302 : VRGKWSWLVPPLSPATTPLWSRRYRAREESPRFVRARCPFHTPTVHASTGHAPTG--------------------
miNOS  451 : CPADWIWLVPPVSGSITPVFHQEMLNYVLSPFYYYQIEPWKTHIWQNEKLRPRRR-EIRFRVLVKVVFFASMLMR
hnNOS  677 : CPADWVWIVPPMSGSITPVFHQEMLNYRLTPSFEYQPDPWNTHVWKGTNGTPTKRRAIGFKKLAEAVKFSAKLMG
beNOS  443 : CPADWAWIVPPISGSLTPVFHQEMVNYILSPAFRYQPDPWKGSATKGAGITRKKT----FKEVANAVKISASLMG

bcNOS    - : ---------------------------
bsNOS    - : ---------------------------
SANOS    - : ---------------------------
deiNOS   - : ---------------------------
miNOS  525 : KVMASRVRATVLFATETGKSEALARDL
hnNOS  752 : QAMAKRVKATILYATETGKSQAYAKTL
beNOS  514 : TLMAKRVKATILYASETGRAQSYAQQL
```

Supplementary Figure 1. Sequence alignment of the bacterial NOS-like proteins and three mammalian oxygenase domains. The sequences shown are (Genebank accession numbers between parenthesis): *Bacillus cereus* NOS -bcNOS (**ABS24145**), *Bacillus subtilis* NOS – bsNOS (**CAB12592**), *Staphylococcus aureus* NOS – SANOS (**BAF78782**), *Deinococcus*

radiodurans NOS –deiNOS (**ABF44571**), murine macrophage iNOS –miNOS (**M84373**), human brain nNOS –hnNOS (**LO2881**) and bovine eNOS –beNOS (**M89952**). The alignment was performed using Clustal W (30). Degree of shading is indicative of sequence homology.

Sequence alignment of the bcNOS protein with prokaryotic and eukaryotic NOS enzymes showed that the residues involved with heme binding in saNOS, bsNOS and the mammalian NOS proteins are conserved in bcNOS. As well, the highly conserved glutamic acid residue found in mammalian NOS proteins to be important for the binding of L-arginine in the substrate-binding site is also conserved in bcNOS.

Expression and purification of bcNOS. Escherichia coli strain BL21(DE3) pLysS transfected with the bcNOS plasmid and grown in Luria-Bertani broth containing 100 µg/mL ampicillin, and induced at an OD (600 nm) of 0.6 with 1 mM IPTG for 4 h. The cells were harvested by centrifugation and stored at –80ºC. The temperature was maintained at 4ºC throughout the purification. The harvested cells were resuspended by adding 2 mL/g of cells buffer A (50 mM Tris-HCl buffer pH 7.0, 1 mM β-mercaptoethanol, 1.0 M NaCl) containing 0.1 mg/mL DNaseI and 0.55 mg/mL lysozyme, protease inhibitors pepstatin 2 µg/mL, bestatin 10 µg/mL, chymostatin 10 µg/mL, and leupeptin 25 µg/mL and stirred until the frozen pellet was dissolved. The cells were lysed using a homogenizer and the lysate was centrifuged at 48,000 g. The cleared lysate was loaded onto Ni^{2+}-agarose resin (Novagen, Madison, WI) equilibrated with tris-imidazole buffer containing 1 M NaCl, and eluted with increasing concentrations of imidazole, always in the presence of 1 M NaCl. Fractions were analyzed by SDS–polyacrylamide electrophoresis and Coomassie blue staining, the fractions containing essentially pure bcNOS protein were pooled, dialyzed against 50 mM Tris-HCl, pH 7.5 and 100 mM NaCl and 10% glycerol, The protein was dialyzed over night at 4°C with the dialysis buffer changed

once after 3 hours. Protein concentration was determined by the Lowry dye binding microassay method using bovine serum albumin as the standard. All assays were performed in quadruplicate.

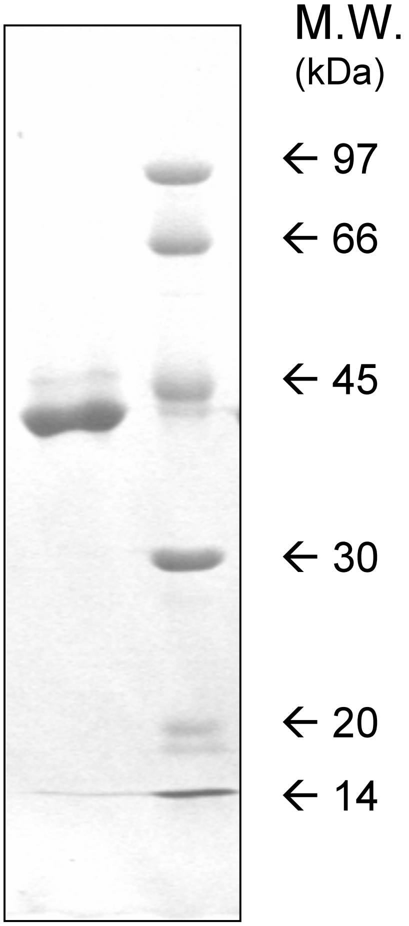

Supplementary Figure 2. SDS-PAGE analysis of purified bcNOS expressed in *Escherichia coli*. Purified bcNOS was electrophoresed on a 10% SDS-PAGE gel (left lane). Comparison with low molecular weight standard in the right lane shows that the approximate molecular weight for bcNOS is 43 kDa.

Difference spectroscopy

Difference spectroscopy was used to measure the binding affinities of imidazole, NOHA, and L-arginine as previously described [see reference 7 in the manuscript]. All experiments were performed at 25 ºC. The bcNOS enzyme was diluted to a final concentration of 10 μM with 50 mM Tris-HCl, pH 7.5 and 10 % glycerol before titration with imidazole to a final concentration of 10 mM. The additions of imidazole caused < 2% volume changes to the sample. Spectra were recorded from 350 nm to 500 nm after each addition. The apparent binding constant, K_d, for imidazole was determined from the x-intercept of a double-reciprocal plot of the difference in the respective peak to trough absorbances versus the imidazole concentration. Linear regression analysis of titration data gave a R^2 value of 0.975. The binding affinities of NOHA and L-arginine were measured after incubation of the bcNOS protein in the presence of 10 mM imidazole for 10 minutes at 25 ºC. Titration of either NOHA or L-arginine caused < 6.5% or < 2% volume changes to the sample, respectively. A double reciprocal plot of the NOHA binding data gave an apparent binding constant, K_{obs}, for NOHA, which was then used to calculate the spectral binding constant, K_s, for NOHA using equation 1.

$$K_s = K_{obs} / (1 + \{[\text{imidazole}]/K_d[\text{imidazole}]\}) \qquad (1)$$

This analysis was also performed on the spectral data obtained by the binding of L-arginine to bcNOS in order to determine the K_s for L-arginine.
